# The Saskatchewan rural health study: an application of a population health framework to understand respiratory health outcomes

**DOI:** 10.1186/1756-0500-5-400

**Published:** 2012-08-01

**Authors:** Punam Pahwa, Chandima P Karunanayake, Louise Hagel, Bonnie Janzen, William Pickett, Donna Rennie, Ambikaipakan Senthilselvan, Josh Lawson, Shelley Kirychuk, James Dosman

**Affiliations:** 1 Canadian Centre for Health and Safety in Agriculture, Royal University Hospital, University of Saskatchewan, 103 Hospital Drive, Saskatoon, SK, S7N 0W8, Canada; 2 Department of Community Health and Epidemiology, University of Saskatchewan, Health Science Building, 107 Wiggins Road, Saskatoon, SK, S7N 5E5, Canada; 3 Department of Community Health and Epidemiology, Queen’s University, Carruthers Hall, Kingston, ON, K7L 3N6, Canada; 4 Accident Research Centre, Monash University, Building 70, Victoria, 3800, Australia; 5 College of Nursing, University of Saskatchewan, 107 Wiggins Road, Saskatoon, SK, S7N 5E5, Canada; 6 Public Health Sciences, School of Public Health, University of Alberta, 3-50 University Terrace, 8303-112 Street, Edmonton, AB, T6G 2T4, Canada

**Keywords:** Rural, Respiratory, Asthma, Chronic bronchitis, Population health framework, Pulmonary function, Contextual

## Abstract

**Background:**

Respiratory disease can impose a significant burden on the health of rural populations. The Saskatchewan Rural Health Study (SRHS) is a new large prospective cohort study of ages 6 and over currently being conducted in farming and non-farming communities to evaluate potential health determinants associated with respiratory outcomes in rural populations. In this article, we describe the rationale and methodology for the adult component.

The study is being conducted over 5 years (2009–15) in two phases, baseline and longitudinal. The *baseline survey* consists of two components, adults and children. The adult component consists of a questionnaire-based evaluation of individual and contextual factors of importance to respiratory health in two sub populations (a *Farm Cohort* and a *Small Town Cohort)* of rural families in Saskatchewan Rural Municipalities (RMs). Clinical studies of lung function and allergy tests are being conducted on selected sub-samples of the two cohorts based on the positive response to the last question on the baseline questionnaire: “Would you be willing to be contacted about having breathing and/or allergy tests at a nearby location?”. We adopted existing population health theory to evaluate *individual factors, contextual factors*, and principal *covariates* on the *outcomes* of chronic bronchitis, chronic obstructive pulmonary disease, asthma and obstructive sleep apnea.

**Findings:**

Of the RMs selected to participate, 32 (89%) out of 36 RMs and 15 (94%) out of 16 small towns within the RMs agreed to participate. Using the mail out survey method developed by Dillman, we obtained completed questionnaires from 4264 households (8261 individuals). We obtained lung function measurements on 1609 adults, allergy skin test information on 1615 adults; both measurements were available on 1549 adults. We observed differences between farm and non-farm rural residents with respect to individual, contextual factors and covariates.

**Discussion:**

There are differences between farm and non-farm rural residents with respect to individual and contextual factors and other variables of importance. The findings of the SRHS will improve knowledge of respiratory disease etiology, assist in the development and targeting of prevention programs, and in planning health services with farm and small town populations.

## Findings

### Background

Of all Canadian provinces, Saskatchewan has a one of the largest proportion of rural dwelling people (35%) [1,2]. An increased prevalence of respiratory symptoms and decreased lung functions have been observed in a diverse group of male farmers compared to rural-dwelling non-farming male control subjects [3]. While much of this research has focused on swine and poultry workers [4-17], less is known about the respiratory status of more general populations of farming and non-farming rural dwellers. It is known that farmers are exposed routinely to organic dusts, pesticides, diesel fumes, welding fumes and gases which increase their risks for acquiring respiratory health problems compared to other non-farming dwellers in rural areas [18-20]. In addition, unlike many other occupations, farming is an occupation where the workplace often overlaps with a residential environment. Accordingly, family members of farmers have increased opportunities for exposure to respiratory hazards, either directly or indirectly. Possible health effects of these exposures are not well documented for many farming and non-farming rural populations. We developed the Saskatchewan Rural Health Study (SRHS) to address these gaps in knowledge.

The theoretical basis for the SRHS is the Population Health Framework (PHF) [21]. This framework provides a structure by which *individual* and *contextual* factors can be studied as possible determinants of respiratory health in farming and non-farming populations. A recent cohort study in our province has successfully utilized and tested this framework to evaluate the etiology of farm injuries [22]. *Individual* and *contextual* factors that could possibly lead to adverse respiratory health outcomes were identified based on historical evidence, both from our own research group and others [23-33].

Past research has demonstrated a relationship between exposure to pesticides and asthma in male farmers [23] and in farm women [24], which raises the possibility of a bystander effect and of gender-specific [9,25] effects of exposures. Grain dust is made up of a number of components including grain parts and bacterial products, which may be involved in the genesis of lung dysfunction in grain dust exposures [26,27]. Respiratory outcomes are also related to bacterial products associated with dampness in homes [28-30], particularly in rural-dwelling women [31].

It has been shown that obesity is an issue in rural communities and is associated with reductions in pulmonary function that may affect women and men differently [25,32,33]. Thus, evidence suggests that determinants of respiratory health may be important for both farming and non-farming rural dwellers, that effects may be gender-specific, and that family members may be affected.

Although some evidence exists, additional evidence using a longitudinal survey approach is required to assess possible determinants of respiratory health among rural farming and non-farming people. In addition, most existing studies have been developed in the absence of an over-riding theory. Population health theory is one contemporary method from which to guide the systematic exploration of how *individual* and *contextual* risk factors influence respiratory health outcomes. To date, the respiratory health status and the determinants of respiratory health in farmers and their families and other rural dwellers have not been well established. The objective of this report is to describe the rationale, methodology, and descriptive results for a new cohort study of respiratory health among adults in rural Saskatchewan. The study aims to address observed gaps in the literature through the application of population health theory.

### Farm and non-farming cohorts

The rural population is defined as consisting of those persons “living in towns and municipalities outside the commuting zone of larger urban centers with a population of 10,000 or more [34].” There are 50,598 farm enterprises in Saskatchewan encompassing 123,385 household residents [35].

Farm residence is defined as an area of land and its buildings (excluding the farm house) which normally produce agricultural commodities intended for sale. Non-farm residence is defined as a home on land that was not used for farming. Designation of residence in a rural dwelling and further classified as living on farm or non-farm based on the question ‘Where is your home located?’ with options: Farm, In town, Acreage. Town and acreage were combined to create a non-farm category, and this categorization was necessary because farming exposures are unique compared to non-farming exposures among rural residents.

## Materials and methods

### Study design for adult baseline survey

The SRHS design is a prospective cohort study being conducted in two phases, using a baseline survey and a five year follow-up study, each examining two study populations. At present, the baseline survey information has been collected. The baseline survey for adults consisted of three stages. The first stage involved recruitment of populations in rural municipalities (RMs) and small towns. The second stage consisted of administration of a mail household questionnaire to the target population. In the third stage, a sub-population participated in clinical assessments that involved anthropometric measures, lung function measurements, and allergy testing. The SRHS was conducted with the understanding and the informed consents of the all participants. The study was approved by the Biomedical Research Ethics Board of the University of Saskatchewan, Canada.

### Stage 1: community recruitment

**Figure 1:**
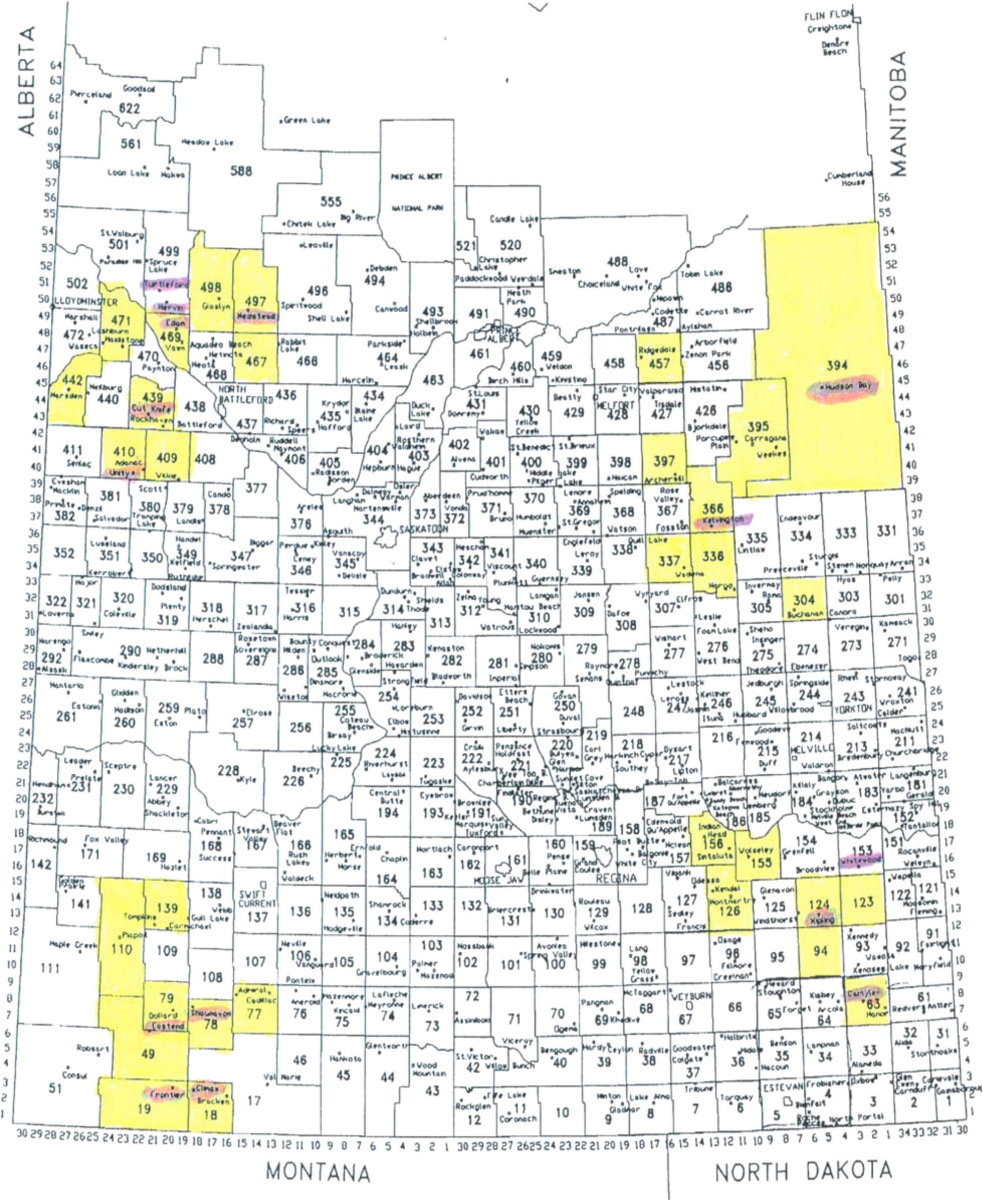
Rural municipalities located in the four study quadrants of the SRHS Study.

The southern half of Saskatchewan is organized into 297 RMs, each governed by an elected council which has the authority to levy taxes applied to landowners. Located within each of the RMs are incorporated towns and villages governed by an elected council that also have the authority to levy taxes. The study base consisted of tax paying households located in RMs and small towns in rural Saskatchewan. A multistage, stratified sampling strategy was used. First, the southern half of the province was divided into four quadrants (Northwest, Northeast, Southwest and Southeast) representing the diverse landscapes and industries in the province (Figure 1). Using the Statistics Canada definition of rural, a sector in each quadrant was identified for inclusion if it was located at least 60 kilometers from an urban center as defined by Statistics Canada [34]. A block of 12 adjacent RMs in each quadrant was identified. Selected RMs that had recently participated in another large cohort study [22] were excluded to avoid low participation rates due to study fatigue and in their place, adjacent RMs were selected.

Purposeful samples of 48 (12 from each quadrant) of the 297 rural municipalities (RMs) and 16 of the 145 towns (usual population 500 to 5000) in Saskatchewan were selected to participate in the study. A sample of 9 RMs was randomly generated for each quadrant. A member of the research team attended a regular meeting of each of the municipal councils for the 36 RMs and 16 small towns to recruit support for the project. The local councils for 32 (89%) [9 from the Northwest, 8 from each of the Northeast and Southwest, and 7 from Southeast] of 36 RMs agreed to participate on behalf of their residents and supplied mailing addresses. Fifteen (94%) [6 from the Northwest, 2 from the Northeast, 4 from Southwest, and 3 from Southeast] of 16 towns agreed to participate on behalf of their residents and also supplied mailing addresses. After excluding ineligible households (e.g. addresses unknown or outside study area, duplicates, deceased) (n = 978), surveys were sent to 11,004 households.

### Stage 2: household recruitment

A registry of mailing addresses was compiled from the taxation lists provided by the rural municipal and small town councils. Based on our sample size calculations to test associations, and assuming a 30% response rate based on our previous study of RM populations [22] we sampled a population of 11,982 tax paying households (approximately 3000 per quadrant). A modified version of The Dillman Total Design Method for Mail and Telephone Surveys [36,37] was used in the administration of a baseline survey; this method was applied in order to maximize response rates. The Dillman method involves a series of mail contacts with all prospective participants. Mailings were addressed personally and sent via first class mail to all households in the database. Duplicate addresses and absentee home owners (those with addresses outside the study areas) were excluded. Study packages contained a letter of invitation, an information pamphlet, and the baseline questionnaire so that recruitment and data collection occurred simultaneously. A key informant in each household was asked to provide household level information and then to complete a section for each adult living in the household.

### Questionnaire development

**Figure 2:**
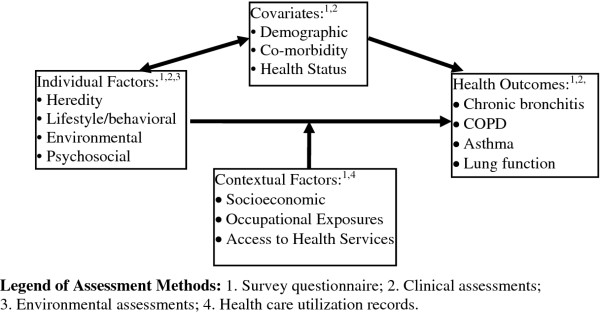
Conceptual Framework based on Population Health Framework of Health Canada (Diagram courtesy of Dr Will Pickett).

A panel consisting of the SRHS research team and two community members (one from a RM and one from a small town) developed the study questionnaire. The questionnaire was designed to include key measures required to test the population health framework (PHF) [21]. According to the PHF *individual* and *contextual* factors and the interaction between them may produce varying levels of risk for adverse respiratory health *outcomes*. More specifically, the SRHS aims to simultaneously evaluate *individual* factors (air quality, cigarette smoke, childhood chest infections, obesity), *contextual factors* (socio-economic, occupational history, past exposures, access to health services), and principal *covariates* on the respiratory health *outcomes* of chronic bronchitis, asthma, chronic obstructive pulmonary disease (COPD), and lung function measurements (see Figure 2). *Covariates* under study include demographic variables, self-perceived health status and co-morbid conditions, due to their association with an increased prevalence of chronic bronchitis, COPD, asthma and decreased lung function values [38].

A pilot study, described elsewhere [39], was conducted to optimize the content and administration of the baseline questionnaire. The RM and a small town which were used for the pilot study were not included in the baseline study. Based on the pilot project responses, several questions were modified in the questionnaire to be used for the baseline survey.

### Stage 3: clinical assessments

The final question on the baseline questionnaire was ‘Would you be willing to be contacted about having breathing and/or allergy tests at a nearby location?’ Those who responded positively to this question were sent a letter of invitation to participate in a clinical assessment. Research nurses trained in spirometry and allergy assessment and located in each study quadrant telephoned each household of consenting participants to arrange a time and a place (usually no greater than 60 kilometers from their residence) for this clinical assessment. Mobile clinics were set up in small towns located in the study area. Clinical measurements included the measurement of height and weight, blood pressure, forced expiratory capacity and allergy skin prick tests for six allergens. The protocol used to obtain these measurements is described below.

### Baseline clinical measurements

Pulmonary function testing was conducted to obtain measures of forced expired volume in one second (FEV_1_), forced vital capacity (FVC), FEV_1_/FVC ratio, and maximum mid-expiratory flow rate (FEF_25-75_). *Sensormedics* (Anaheim, CA) dry rolling seal spirometers were used for pulmonary function testing [9,40,41]. Measurements were taken according to standards of the American Thoracic Society criteria [42]. Percent predicted values for pulmonary function test variables were equated from the regression equations of Crapo et al. [43]. Allergy skin prick testing (SPT) was conducted using the skin prick method with a panel of six non-food allergens and two controls. Antigens used for testing were *alternaria*, house dust mite, *cladosporium*, local grasses, wheat dust, and cat dander. A positive (histamine) and negative (diluent) controls were used by methods as previously described [44]. Standardized allergen extracts were used as recommended by the Academy of Allergy, Asthma and Immunology [45]. SPT was performed according to the recommended protocol of practice parameters for Allergy, Asthma and Immunology and the American College of Allergy, Asthma and Immunology [45]. Subjects were considered positive for atopy if there was with a raised wheal greater than 3 mm compared to the saline control for one or more allergens tested. Quality of the lung function measurements data and skin prick testing was good as all spirometry measurements and skin prick testing were conducted by trained registered nurses.

### Variables of interest

#### Outcomes

The information on respiratory health *outcomes* of asthma, chronic bronchitis, and chronic obstructive pulmonary disease (COPD) was ascertained from the survey questionnaire based on the following questions:

##### For asthma

B-22 Have you ever had asthma?

Yes

No - > If no, go to question B-26.

B-23 If Yes to B-22:

Do you still have it? Yes No

Was it confirmed by a doctor? Yes No

At what age did it start? ____ age in years

If you no longer have it, at what age did it stop?

____ age in years

B-24 If yes to B-22, how many times have you required services for asthma from the following places during the past 12 months?

Hospital inpatient: _________ times

Emergency room outpatient: _________ times

Doctor’s office: _________ times

B-25 If yes to B-22, which of the following statements best describes your asthma medication use in the past 12 months

.Never in the past 12 months

.At least once in the past 12 months

.At least once per month

.At least once per week

.Every day

##### For chronic bronchitis and COPD

B-51 Has a doctor ever said you had any of the following chest illnesses:

Chronic Bronchitis

COPD (Chronic Obstructive Pulmonary Disease)

The information on COPD was ascertained from the questionnaire and as well as from the clinical pulmonary function tests.

#### Contextual factors

The contextual factors associated with respiratory health outcomes of interest in this study were residence location, socioeconomic status and indoor environment.

(i) Designation of residence in a rural dwelling and further classified as living on farm or non-farm based on the question ‘Where is your home located?’. Details were given in the section ‘Farm and non-farming cohort’.

(ii) Socioeconomic status was assessed using two questions. They were

Household Income adequacy was a derived variable with four categories based on various combinations of total household income and the number of people living in the household according the Statistics Canada definition [46]. The description of the categories is given below.

The *lowest income adequacy* category consisted of income less than $15,000 and household size of 1 or 2 persons, or total household income less than $20,000 and household size of 3 or 4 persons, or total household income less than $30,000 and household size of 5 or more persons. The *lower middle income* category consisted of total household income between $15,000-$29,999 and household size of 1 or 2 persons, or total lower income less than $20,000-$39,999 and household size of 3 or 4 persons, or total household income between $30,000-$59,999 and household size 5 or more persons. The *upper middle income* category consisted of total household income between $30,000-$59,999 and household size of 1 or 2 persons, or total lower income less than $40,000-$79,999 and household size of 3 or 4 persons, or total household income between $60,000-$79,999 and household size 5 or more persons. The *highest income* adequacy category consisted of $60,000 or more and household size of 1 or 2 persons, or total income category was $80,000 or more and household size of 3 persons or more.

(a) What is your best estimate of the total income, before taxes and deductions, of all household members from all sources in the past 12 months?

.Less than $14,999

.$15,000 to $19,999

.$20,000 to $29,999

.$30,000 to $39,999

.$40,000 to $49,999

.$50,000 to $59,999

.$60,000 to $79,999

.$80,000 or more

(b) At the end of the month, how much money do you have left over? (Please check only one)

.Some money

.Just enough money

.Not enough money

(iii) Indoor environment was assessed by response to questions about dampness, mold, smoking inside the house, number of people and bedrooms, air conditioning, pets in home, pesticides applied inside home and fuel source. Information on dampness, mold and smoking inside the house was collected by the following three questions respectively: “During the past 12 months, has there been water or dampness in your home from broken pipes, leaks, heavy rain, or floods? (Yes/No/Don’t know)”; “Does your home (including basement) frequently have a mildew odor or musty smell? (Yes/No/Don’t know)”; “Do any of the people who live in your house use any of the following tobacco products in the home? (Cigarettes: Yes/No/Don’t know; Cigars: Yes/No/Don’t know; Pipes: Yes/No/Don’t know)”.

(iv) Information about the remaining contextual factors was collected using the following questions: “How many people live in your home? (This variable includes all persons including children who usually live in home)”, “How many bedrooms do you have in your home?”, “Does your home have air conditioning?”, “In the past 12 months have you had any pets living in your home”, “Within the past 12 months, were pesticides applied inside your residence (e.g. raid, spider bait, any bait, rat bait), and “Is natural gas primary fuel source to heat your home?”.

(v) In addition information about access to health care in the past 12 months were collected using following questions: “Do you and your family members in your household have access to a regular family doctor or nurse practitioner?”, In the past 12 months did you ever experience any difficulties getting the routine or on-going care for you or a family member in your household ”, “In the past 12 months, have you required a visit to a medical specialist for a diagnosis or consultation for yourself or a family member in your household?”, “In the past 12 months did you ever experience any difficulty getting the specialist care you needed for a diagnosis or consultation for yourself or a family member in your household?”, “In the past 12 months, have you or a family member in your household required immediate 24 hour health care services for a medical emergency?”, and “In the past 12 months, did you ever experience any difficulties getting immediate 24 hour health care services for a medical emergency for yourself or a family member in your household?”.

#### Individual factors

The individual factors considered were: (i) individual educational attainment. The highest level of education (less than high school, completed high school, completed university, completed post-secondary education other than above) recoded into a new variable “Education” combining the first two levels (≤ grade 12) and last two levels (> grade 12) for the analysis; (ii) lifestyle or behavior-related factors with an expected impact on health including smoking and physical activity; (iii) the general health status and co-morbid conditions such as diabetes, heart disease, heart attack, hardening of the arteries, high blood pressure and cancer; (iv) family history of respiratory health problems; among first degree relatives (father, mother, brother/sister) excluding offspring; and (v) environmental and occupational exposures. Information about environmental and occupational exposures was based on the question; “Have you ever been exposed to any of the following in your workplace? [the list included: grain dust, mine dust, asbestos dust, wood dust, other dust ___specify, livestock, smoke from stubble burning, diesel fumes, welding fumes, solvent fumes, oil/gas well fumes, herbicides (to kill plants), fungicides (to treat grain), insecticides (to kill insects), molds, radiation, Other __ specify]; information was also collected on the frequency (Daily, Weekly, Monthly, Occasionally) and duration “How many years?” of usage.

#### Covariates

Information was obtained on covariates of importance such as age, sex, marital status, co-morbid conditions (allergy) and body mass index. Body mass index was derived from self-reported weight and height of the respondent. Response to the question “Have you ever had an allergic reaction to any of the following: house dust; cats; dogs; grasses; pollens; molds; other (specify)?” was used to assess allergic conditions.

#### Sampling frame

In order to accomplish the objective, we proposed to use a Sampling Frame of 5000 farm residents and 4000 Small Town residents living in isolated rural areas of four quadrants of the province.

Based on our experience in the Farm Injury Cohort Study [22], we conservatively predict a 65% response rate among the 5000 members of the Farm Cohort to have 3250 respondents to participate in the mail baseline questionnaire survey (Table 1). We proposed that the Small Town Cohort will be comprised of persons dwelling in small rural Saskatchewan communities, selected on a regional basis, with a collective population of 4000 persons. Of these we anticipated, conservatively, that *~65%,* or 2600 persons would respond to the mail baseline questionnaire survey.

**Table 1:** Proposed and Observed number of participants in Farm Cohort and Small Town Cohort for Baseline Study

	**Farm Cohort**		**Small Town Cohort**	
	**Proposed sample**	**Observed sample**	**Proposed sample**	**Observed sample**
Self-report questionnaire	3250	4472	2600	3785
Clinical measurements (25%)	813	Lung function, n = 930	650	Lung function, n = 679
		Allergy test, n = 929		Allergy test, n = 686
		Both, n = 896		Both, n = 653

### Sample size calculations

#### Dichotomous outcome

To compare prevalence of a respiratory condition between two groups, the sample size [47,48] required to test the hypotheses was calculated based on the following modified formula [49]:

(1) $$n={\left[\frac{z_{a}\sqrt{2\bar{p}}\left(1-\bar{p}\right)+z_{\beta }\sqrt{p_{1}\left(1-p_{1}\right)+p_{2}\left(1-p_{2}\right)}}{d}\right]}^{2}*\left[1+\left(m-1\right)\rho \right]$$

where *p*_1_ (prevalence of respiratory condition in *the Farm Cohort*) and *p*_2_ (prevalence of respiratory condition in the *Small Town Cohort*) are pre-study estimates of the two proportions to be compared, *d* = |*p*_1_ - *p*_2_| (i.e., the minimum expected difference), $\bar{p}$= (*p*_1_ + *p*_2_)/2, and we require *n* subjects per group for type-I error (α) = 0.05 and power (1-β) = 0.80. The adjustment was incorporated by using the variance inflation factor (*VIF*) to account for clustering within household, where *VIF* = 1+ (m-1)*ρ (where m = number of individuals per household assumed to be 2.4, and *ρ* = intra-class correlation coefficient for within subject clustering = 0.3, 0.5). The required sample sizes for selected values of p_1_, p_2_ are given in Additional file 1 Table S1. The proposed sample sizes of 3250 and 2600 in each of the groups are adequate for comparison in the total sample (see Table 1).

#### Continuous outcomes

To compare continuous respiratory outcome between two groups, the sample size [50,51] required to test the hypotheses was calculated based on the following modified formula [49]:

(2) $$n=\frac{2*{\left(z_{a}+z_{\beta }\right)}^{2}\sigma^{2}}{d^{2}}*\left[1+\left(m-1\right)\rho \right]$$

To detect the smallest meaningful difference between the means of two groups, ‘d’ (e.g. *Farm Cohort* vs. *Small Town Cohort*), for a given standard deviation (assumed to be equal for both groups), σ, we require ‘*n’* subjects per group for type-I error (α) = 0.05 and power (1-β) = 0.80. The required sample sizes for selected values of μ_1_, μ_2_, σ to detect a minimum difference of ‘d’ between two groups to achieve 80% power are given in Additional file 2 Table S2. The adjustment was incorporated by using the variance inflation factor (*VIF*) to account for clustering within household, where *VIF* = 1+ (m-1)*ρ (where m = number of individuals per household assumed to be 2.4, and *ρ* = intra-class correlation coefficient for within subject clustering = 0.3, 0.5). The proposed sample sizes of 3250 and 2600 in each of the groups are adequate for comparison in the total sample.

### Statistical analysis

Response rates were first examined descriptively by rural RM and small town categories. Descriptive results comparing the characteristics of farming (living on a farm) and non-farming (living in a small town or on an acreage) populations are presented in this report. Statistical analysis was completed using IBM SPSS Statistics Version 19 (IBM Corporation, Armonk, New York). For baseline data, standard classical techniques, such as *χ*^2^ analysis techniques, t-tests and analysis of variance were utilized for descriptive group comparisons. Such comparisons were conducted for *individual* characteristics (e.g. age, income, smoking status), *contextual* factors (e.g. household income, number of people living in the household), other *covariates* and respiratory *outcomes*. The main purpose of this analysis was to demonstrate that we had sufficient variability in exposures, covariates and outcomes to achieve our main analytical goals.

## Results

Of the RMs and small towns selected to participate, 32 (89%) out of 36 RMs and 15 (94%) out of 16 small towns agreed to participate. Questionnaires were mailed to 11,982 households located in four geographical regions (Northwest, Southwest, Southeast, and Northeast) of Saskatchewan, Canada in 2010. Of these, 978 addresses were excluded because ratepayers were not living in the area, they had moved, were deceased or address was a duplication or unknown.

Data were entered and cleaned on an ongoing basis. Final response rates are presented in Table 2. The response rate to the mail-out survey was moderate (42%), although it is uncommon to get a higher response rate for mail-out surveys without inducement. The participation rate for non-farm dwellers and farm dwellers were similar, 42.2% and 41.9% respectively. We obtained completed questionnaires from 4624 households (8261 individuals). Lung function measurements were completed for 1609 adults and allergy skin tests information was available for 1615 adults. Both measurements were available for 1549 adults. There were a significantly higher proportion of individuals older than 65 years in the north east quadrant compared to other quadrants (Table 3). Compared to north east quadrant, a higher proportion of individuals lived in non-farm locations in the other three quadrants (Table 3). Baseline descriptive results for selected individual and household factor, and selected covariates stratified by farm and non-farm locations are provided in Additional file 3 Tables S3 and Additional file 4 Table S4.

**Table 2:** Response rates achieved in Saskatchewan Rural Health Study in the 2010 baseline survey

**Response to Mail Survey**	**Small Town**	**RM**
	**(n = 15)**	**(n = 32)**
Household addresses (ratepayers) ^#^	**5318**	**5683**
(n = 11004)		
Household Returned Surveys, n (%)	2800 (52.7)	2910 (51.2)
No Response, n (%)	2518 (47.3)	2773 (48.8)
Response rate (based on household addresses)	2242 (42.2)	2380 (41.9)
(n = 4624), n (%)		
(Note: Two households were not identified)		
Persons participating (n = 8261)	**3785**	**4472**
(Note: four individuals were not identified by town/RM)		
Age (mean ± SE)	56.3 ± 0.28	55.9 ± 0.22
Male : Female ratio	1774/2007	2292/2179
	0.88	1.05
Clinical assessments (n = 1675)		
Lung function, n (%)	679 (40.4)	930 (55.3)
Allergy Test, n (%)	686 (40.8)	929 (55.3)
Both, n (%)	653 (38.8)	896 (53.3)

^#^ Town/RM status was not identified in three households.

**Table 3:** Populations characteristics by quadrant

**Characteristics**	**Quadrant**^#^				**P value**
	**South West**	**South East**	**North East**	**North West**	
	**N = 878**	**N = 1010**	**N = 1315**	**N = 1419**	
	**n = 1538**	**n = 1792**	**n = 2400**	**n = 2527**	
Rural Municipality, n (%)					
Town	691 (44.9)	921 (51.4)	1622 (67.6)	1238 (49.0)	<0.0001^***^
RM	847 (55.1)	871 (48.6)	778 (32.4)	1289 (51.0)	
Location of Home, n (%)					
Farm	552 (36.0)	704 (39.6)	1192 (50.0)	997 (39.7)	<0.0001^***^
Non-Farm	980 (64.0)	1072 (60.4)	1193 (50.0)	1514 (60.3)	
Age group, yrs, n (%)					
18–45	341 (22.2)	464 (25.9)	485 (20.2)	647 (25.6)	<0.0001^***^
46–55	433 (28.2)	441 (24.6)	552 (23.0)	620 (24.6)	
56–65	319 (20.8)	422 (23.5)	599 (25.0)	608 (24.1)	
>65	444 (28.9)	465 (25.9)	762 (31.8)	650 (25.7)	
Sex, n (%)					
Male	750 (48.8)	886 (49.4)	1201 (50.0)	1229 (48.7)	0.791
Female	788 (51.2)	906 (50.6)	1199 (50.0)	1293 (51.3)	

# *N* = Number of households; *n* = number of individuals; Four individuals in 2 households could not identified.

^*^p<0.05 ^**^P<0.01 ^***^P<0.0001.

### Comparison of individual factors and covariates

A higher prevalence of current smokers was found among *non-farm* population compared to the *farm* population, (13.5% vs. 9.5% respectively). A higher proportion of the *farm* population reported very good (38.3% vs. 33.2% respectively) to excellent (9.5% vs. 8.5% respectively) self-perceived health compared to the *non-farm* population. A significantly higher proportion of *non-farm* dwellers reported the presence of co-morbid conditions compared to the *farm* dwellers (diabetes: 10.9% vs. 7.1%; heart disease: 8.9% vs. 5.7%; heart attack: 5.2% vs. 3.0%; hardening of the arteries: 3.9% vs. 2.8%; high blood pressure: 36.1% vs. 30.2%; cancer: 8.8% vs. 7.6%). Compared to the *non-farm* dwellers, a significantly higher proportion of *farm* dwellers were exposed to various occupational exposures (grain dust: 86.7% vs. 54.6%; wood dust: 45.4% vs. 33.5%; livestock: 69.6% vs. 37.9%; smoke stubble: 51.7% vs. 31.7%; diesel fumes: 72.5% vs. 48.5%; welding fumes: 52.8% vs. 32.5%; solvents: 39.7% vs. 32.2%; pesticides including herbicides: 66.6% vs. 38.9%; fungicides: 44.3% vs. 24.4%; insecticides: 56.3% vs. 36.7%; and molds:46.3% vs. 25.9%).

### Comparison of contextual factors

A higher proportion of households on *farm* locations had 2 or more people (28.0%) and 3 or more bedrooms (44.2%) compared to households on *non-farm* locations (≥ 2 people - 22.9%, and ≥ 3 bedrooms-32.6%). Dampness and mildew odor were significantly more prevalent in houses located on farms compared to those on non-farms. The proportion of pesticides applied inside home was significantly higher in houses located on farms compared to those on non-farms. The proportion of use of tobacco in homes was higher in houses located on non-farm locations compared to those located on farms. Three household income variables (Total household income, household income adequacy, and Money left at end of month) were significantly related to the residence status of farm/non-farm. It is observed that higher proportion of farm households reported highest income.

## Discussion

The SRHS is a large, prospective cohort study based on a modified conceptual framework of the PHF that was successfully used as a framework for a related injury cohort study [22]. The purpose of the SRHS is to test the hypothesis that rural environments, as determinants of health, are associated with respiratory outcomes in farming and non-farming rural people. To our knowledge, no other Canadian study has been conducted to investigate the health determinants of respiratory health among rural people in this manner. Hence, the results of this study will contribute to understanding the health determinants of respiratory health status among rural farming and non-farming people. The complex methodological approach used in this study captured many factors associated with variability found in rural populations. Appropriate statistical methods that account for the nested and hence clustered nature of the sampling design (individuals nested within households, nested within RMs) will be utilized to test the major hypothesis for four primary respiratory health *outcomes*: chronic bronchitis, asthma, COPD, and lung function measurements. Based on our preliminary findings, quadrant level, location of home (farm versus non-farm) and sex will be examined in all future analyses.

This manuscript reports the results derived from the baseline data on farming and non-farming adults. The statistical analyses were conducted to compare demographic characteristics, *individual* and *contextual factors*, and important *covariates* among farming and non-farming people. As shown in Additional file 3 Table S3 non-farming residents were significantly older than farming residents in rural Saskatchewan. Fifty-two percent of residents were males in farming locations compared to 47.2% male residents in non-framing locations. Significantly higher number of farm residents were either married or lived as a common-law, or living together compared to non-farming residents. No difference was observed in the BMI distribution between farming and non-farming residents.

Compared to non-farming residents, a higher proportion of farming residents reported to be in excellent or very good health. A higher proportion of co-morbid conditions (diabetes, heart disease, heart attack, hardening of the arteries, high blood pressure, cancer) were observed in non-farming population.

There is limited literature available for comparison of our results and the definitions of farm and non-farm residents vary. The Wisconsin Rural Women’s Health Study reported that the prevalence of current smoking was significantly higher among non-farm women residents [52]. Also, the same study reported that the prevalence of hypertension and obesity was significantly higher among farm women residents [52]. In contrast to that for both men and women, we observed significantly higher prevalence in current smoking in the non-farm residents. Both non-farm men and women in our study reported significantly higher prevalence of obesity and co-morbid conditions including diabetes, heart disease, high blood pressure, lung disease and cancer.

The Iowa farm and non-farm household study reported greater pesticides exposure among farm residents compared to non-farm residents [53]. Another study of children of Iowa farmers and non-farmers reported a significantly higher pesticide exposure among farm children [54]. We also observed that a higher proportion of farm residents were ever exposed to pesticides (herbicides, fungicides and insecticides). Also significantly a higher proportion of farm-residents applied pesticides inside their homes compared to non-farm residents.

We observed that farm residents had a significantly higher household income level compared to non-farm residents. Similar to this Stiefelmeyer reported that on an average, total farm family income exceeds that of rural non-farm counterparts in Canada [55].

A study examining the importance of place of residence on use of health services observed that nonmetropolitan elderly, both farm and non-farm, make fewer physician visits than do their metropolitan counterparts [56]. Also they reported significantly fewer physician visits for nonmetropolitan farm residents compared to nonmetropolitan non-farm residents [56]. In our study we found no differences in physician visits between farm and non-farm residents.

### Strengths and limitations of the SRHS

There are several major strengths to this study. A large sample size will provide adequate statistical power to test the major statistical hypothesis and several secondary hypotheses to investigate various etiologies of respiratory health, as evident in our descriptive analyses presented here. Extensive information has been obtained on *individual* and contextual factors and important *covariates* via self-administered mail-out questionnaires and clinical and allergy tests on a self-selected group of study participants. The population studied live in widespread locations in the four quadrants of the province representing a wide range of geographical areas in Saskatchewan. Hence a mail questionnaire survey was the best option for us. Other authors have discussed this issue and they have concluded that with the increasing cost of interviewing, a mail questionnaire surveys in widely spread geographical areas was the best [37,57]. Our team consists of researchers from multi-disciplinary areas bringing a comprehensive set of perspectives on the topic of respiratory health in two rural cohorts.

One of the limitations of our study is that the rural areas examined have either no metropolitan influence zone (MIZ), or weak or moderate MIZ. There was no RM or small town with a strong MIZ. This indicates that our study population may not be representative sample of Saskatchewan rural population. Therefore, it is necessary to be cautious in generalizing our results related to the respiratory health outcomes (based on future analyses) to the entire Saskatchewan rural population. However, we may be able to generalize our results for rural areas with no, weak, or moderate MIZ.

Since Saskatchewan remains a considerably rural province, the information resulting from this project will assist in prevention programs and in planning for respiratory health service delivery to rural areas. Identification of factors that promote health and prevent disease in rural populations will help to inform strategies used to improve disease outcomes, including more effective public education programs and more rationally conceived health services delivery strategies. The findings from this study will help to inform policy in Saskatchewan at the Regional Health Authorities level, in addition to helping shape and determine national and provincial health services priorities.

### Future directions of the SRHS

Phase 2, which will commence in 2014, will consist of a longitudinal survey follow-up of individuals who participated in the baseline survey. In order to maintain the high retention rate in our follow-up study, we will be in touch with the study populations, and RM and small town councilors via regular local newsletters, local newspapers, presentation of results at RM and small town council meetings, the project website (under construction) and other effective communication media.

The longitudinal component will consist of a follow-up questionnaire through which we will acquire data on principal *individual* and *contextual* factors of importance to respiratory health in farming and non-farming rural people in Saskatchewan, and clinical studies of principal lung function measurements and allergy tests as outlined above for the baseline survey. In our future articles, based on the Population Health Framework [22], we will test the overall hypothesis that rural environments as determinants of health are associated with respiratory outcomes in rural people.

## Conclusions

There are differences between farm and non-farm rural residents with respect to individual and contextual factors; and other variables of importance. The association of these factors with primary respiratory health outcomes in SRHS might be different in farm and non-farm populations. We will be exploring these associations in our future manuscripts. The findings of the SRHS will improve knowledge of respiratory disease etiology, assist in the development and targeting of prevention programs for rural population of Saskatchewan. The information resulting from this project will assist in planning health services delivery for farm and small town populations.

## Abbreviations

SRHS, Saskatchewan Rural Health Study; RM, Rural Municipality; PHF, Population Health Framework; FEV1, Forced Expired Volume in one second; FVC, Forced Vital Capacity; FEF25-75, Maximum mid-expiratory flow rate; SPT, Skin Prick Testing.

## Competing interests

All authors declare that they have no competing interests.

## Authors’ contributions

All authors contributed to grant writing, development of study design, questionnaire development, and study coordination. JAD and PP are the co-principal investigators of the SRHS study. CPK is the biostatistician and she supervised every stage of data entry and data cleaning and conducted the statistical analyses. LH is the project manager. PP prepared the manuscript and JAD, WP, DR, LH and CPK significantly contributed to manuscript preparation. All authors read and approved the final manuscript.

## Supplementary Material

Additional file 1 Table S1 Sample size required per group for selected values of p_**1**_**, p**_**2**_**and d.** Description: Sample size required per group for selected values of p_1_, p_2_ and d comparing two proportions. Click here for file

Additional file 2 Table S2 Sample size required per group to detect a minimum difference, d between two groups for selected values of σ. Description: Sample size required per group to detect a minimum difference, d between two groups for selected values of σ for comparing continuous outcomes. Click here for file

Additional file 3 Table S3 Comparison of baseline *individual* factors and *covariates* of farm and non-farm people participating in the SRHS. Description: Descriptive comparison of baseline *individual* factors and *covariates* of farm and non-farm people participating in the SRHS. Click here for file

Additional file 4 Table S4 Comparison of household covariates and household factors of farm and non-farm people participating in the Saskatchewan Rural Health Study.Description: Descriptive Comparison of household covariates and household factors of farm and non-farm people participating in the Saskatchewan Rural Health Study. Click here for file
