# Supplementary material for: The Saskatchewan rural health study: an application of a population health framework to understand respiratory health outcomes
Source: BMC Res Notes. 2012 Aug 1;5:400. doi: 10.1186/1756-0500-5-400 (PMC3438108; doi:10.1186/1756-0500-5-400)
Supplement: Additional file 1 Table S1 — Sample size required per group for selected values of p1, p2and d. Description: Sample size required per group for selected values of p1, p2 and d comparing two proportions. [file 1756-0500-5-400-S1.docx]

T**able S1. Sample size required per group for selected values of p_1_, p_2_ and d.**

| Prevalence in *Farm Cohort* (*p_1_*) | Prevalence in Small *Town Cohort* (*p_2_*) | *d* = \|*p*_1_ - *p*_2_\| | Sample size/ group | Sample size/group after adjustment of clustering, ρ=0.3 | Sample size/group after adjustment of clustering, ρ=0.5 |
| --- | --- | --- | --- | --- | --- |
| 0.15 | 0.10 | 0.05 | 685 | 973 | 1165 |
| 0.20 | 0.10 | 0.10 | 199 | 283 | 338 |
| 0.25 | 0.10 | 0.15 | 99 | 141 | 168 |
| 0.20 | 0.15 | 0.05 | 904 | 1284 | 1537 |
| 0.25 | 0.15 | 0.10 | 250 | 355 | 425 |
| 0.30 | 0.15 | 0.15 | 120 | 170 | 204 |
| 0.25 | 0.20 | 0.05 | 1093 | 1552 | 1858 |
| 0.30 | 0.20 | 0.10 | 293 | 416 | 498 |
| 0.35 | 0.20 | 0.15 | 138 | 196 | 235 |
