# Supplementary material for: The Saskatchewan rural health study: an application of a population health framework to understand respiratory health outcomes
Source: BMC Res Notes. 2012 Aug 1;5:400. doi: 10.1186/1756-0500-5-400 (PMC3438108; doi:10.1186/1756-0500-5-400)
Supplement: Additional file 2 Table S2 — Sample size required per group to detect a minimum difference, d between two groups for selected values of σ. Description: Sample size required per group to detect a minimum difference, d between two groups for selected values of σ for comparing continuous outcomes. [file 1756-0500-5-400-S2.docx]

**Table S2. Sample size required per group to detect a minimum difference, d between two groups for selected values of σ .**

| Smallest meaningful difference (*d*) | Given pooled standard deviation (σ) | Sample size/group | Sample size/group after adjustment of clustering, ρ=0.3 | Sample size/group after adjustment of clustering, ρ=0.5 |
| --- | --- | --- | --- | --- |
| 5 | 10 | 63 | 89 | 107 |
| 5 | 15 | 141 | 200 | 240 |
| 5 | 20 | 251 | 356 | 427 |
| 5 | 25 | 392 | 557 | 666 |
| 10 | 10 | 16 | 23 | 27 |
| 10 | 15 | 35 | 50 | 60 |
| 10 | 20 | 63 | 90 | 107 |
| 10 | 25 | 98 | 139 | 167 |
| 15 | 10 | 7 | 10 | 12 |
| 15 | 15 | 16 | 23 | 27 |
| 15 | 20 | 28 | 40 | 48 |
| 15 | 25 | 44 | 62 | 75 |
