# Supplementary material for: The Saskatchewan rural health study: an application of a population health framework to understand respiratory health outcomes
Source: BMC Res Notes. 2012 Aug 1;5:400. doi: 10.1186/1756-0500-5-400 (PMC3438108; doi:10.1186/1756-0500-5-400)
Supplement: Additional file 3 Table S3 — Comparison of baseline individual factors and covariates of farm and non-farm people participating in the SRHS. Description: Descriptive comparison of baseline individual factors and covariates of farm and non-farm people participating in the SRHS. [file 1756-0500-5-400-S3.doc]

**Table S3. Comparison of baseline *individual* factors and *covariates* of farm and non-farm people participating in the SRHS**

| **(a) INDIVIDUAL COVARIATES** | Farm | Non-Farm | P value |
| --- | --- | --- | --- |
| Age (Yrs)  Mean ± SE  Range | 54.9 ±0.2  18-94 | 56.8 ± 0.2  18-101 | <0.0001 |
| Height (cm)  Mean ± SE  Range | 170.6 ± 0.2  91.4 – 208.3 | 169.4 ± 0.1  70 – 200.7 | <0.0001 |
| Weight(Kg)  Mean ± SE  Range | 81.1 ± 0.3  28.0 – 168.2 | 80.6 ± 0.3  29.5 – 181.8 | 0.178 |
|  | n (%) | n (%) |  |
| Sex  Male  Female | 1794 (52.1)  1650 (47.9) | 2246 (47.2)  2514 (52.8) | <0.0001 |
| Marital Status  Married/Common law/  living together  Widowed/ Divorced/  Separated Single, never married | 2997 (87.2)  440 (12.8) | 3743 (79.0)  995 (21.0) | <0.0001 |
| Body Mass Index (BMI)  Normal (<25Kg/m^2^)  Overweight (25-30Kg/m^2^)  Obese (>30 Kg/m^2^) | 526 (30.2)  721 (41.4)  495 (28.4) | 789 (29.7)  1083 (40.7)  787 (29.6) | 0.700 |

| **(b) INDIVIDUAL FACTORS** | Farm | Non-Farm | P value |
| --- | --- | --- | --- |
| **Socioeconomic** |  |
| Education  ≤ grade 12  > grade 12 | 2133 (62.5)  1280 (37.5) | 2776 (59.1)  1921 (40.9) | 0.002 |
| **Lifestyle Factors** |  |  |  |
| Smoking  Never Smoker  Ex-Smoker  Current Smoker | 2048 (59.7)  1056 (30.8)  327 (9.5) | 2250 (47.5)  1846 (39.0)  639 (13.5) | <0.0001 |
| Physical Activity-Do you exercise  Yes  No | 1892 (56.3)  1471 (43.7) | 2700 (58.3)  1931 (41.7) | 0.068 |
| **Health Status and Co-morbid conditions** |  |  |  |
| Perception of health  Excellent  Very Good  Good  Fair  Poor | 324 (9.5)  1308 (38.3)  1367 (40.0)  367 (10.7)  53 (1.6) | 400 (8.5)  1569 (33.2)  1940 (41.1)  676 (14.3)  138 (2.9) | <0.0001 |
| Ever diagnosed with diabetes?  Ever diagnosed with heart disease?  Ever had heart attack?  Ever had hardening of the arteries?  Ever had High blood pressure?  Ever had cancer? | 243 (7.1)  194 (5.7)  101 (3.0)  93 (2.8)  1027 (30.2)  258 (7.6) | 514 (10.9)  414 (8.9)  243 (5.2)  181 (3.9)  1690 (36.1)  413 (8.8) | <0.0001  <0.0001  <0.0001  0.005  <0.0001  0.048 |
| Family History of Lung Diseases(asthma, emphysema, chronic bronchitis) |  |  |  |
| Dad ever had lung trouble | 534 (15.8) | 711 (15.3) | <0.0001 |
| Mom ever had lung trouble | 345 (10.2) | 533 (11.5) | 0.003 |
| Sibling ever had lung trouble | 344 (12.1) | 520 (13.7) | 0.056 |
| **(c) OCCUPATIONAL EXPOSURES** | Farm | Non-Farm | P value |
| Ever been exposed to grain dust at work | 2968 (86.7) | 2524 (54.6) | <0.0001 |
| Ever been exposed to mine dust at work | 169 (4.9) | 274 (5.9) |  |
| Ever been exposed to wood dust at work | 1554 (45.4) | 1550 (33.5) | <0.0001 |
| Ever been exposed to livestock at work | 2383 (69.6) | 1750 (37.9) | <0.0001 |
| Ever been exposed to smoke stubble at work | 1770 (51.7) | 1463 (31.7) | <0.0001 |
| Ever been exposed to diesel fumes at work | 2482 (72.5) | 2243 (48.5) | <0.0001 |
| Ever been exposed to welding fumes at work | 1808 (52.8) | 1500 (32.5) | <0.0001 |
| Ever been exposed to solvents at work | 1361 (39.7) | 1488 (32.2) | <0.0001 |
| Ever been exposed to oil gas fumes at work | 834 (24.4) | 1095 (23.7) | 0.489 |
| Ever been exposed to herbicides at work | 2279 (66.6) | 1798 (38.9) | <0.0001 |
| Ever been exposed to fungicides at work | 1516 (44.3) | 1126 (24.4) | <0.0001 |
| Ever been exposed to insecticides at work | 1928 (56.3) | 1697 (36.7) | <0.0001 |
| Ever been exposed to molds at work | 1587 (46.3) | 1197 (25.9) | <0.0001 |
| Have you ever been exposed to radiation at work | 277 (8.1) | 404 (8.7) | 0.300 |
