# Supplementary material for: The Saskatchewan rural health study: an application of a population health framework to understand respiratory health outcomes
Source: BMC Res Notes. 2012 Aug 1;5:400. doi: 10.1186/1756-0500-5-400 (PMC3438108; doi:10.1186/1756-0500-5-400)
Supplement: Additional file 4 Table S4 — Comparison of household covariates and household factors of farm and non-farm people participating in the Saskatchewan Rural Health Study.Description: Descriptive Comparison of household covariates and household factors of farm and non-farm people participating in the Saskatchewan Rural Health Study. [file 1756-0500-5-400-S4.doc]

**Table S4. Comparison of *contextual* factors of farm and non-farm people participating in the Saskatchewan Rural Health Study**

|  | **Farm** | **Non-Farm** | **P value** |
| --- | --- | --- | --- |
| ***CONTEXTUAL* FACTORS** |  |  |  |
| **Family/household structure** |  |  |  |
| Number of People |  |  |  |
| Mean + SE | 2.4+0.03 | 2.1+0.02 | <0.0001 |
| Range | 1-9 | 1-10 |  |
| Number of Bedrooms |  |  |  |
| Mean + SE | 3.5+0.02 | 3.1+0.02 | <0.0001 |
| Range | 1-8 | 1-9 |  |
|  | n (%) | n (%) |  |
| Own home |  |  |  |
| Yes | 1759 (97.8) | 2582 (94.9) | <0.0001 |
| No | 40 (2.2) | 195 (7.0) |  |
| Number of people in the household  ≤ 2 people  > 2 people | 1301 (72.0)  507 (28.0) | 2144 (77.1)  638 (22.9) | <0.0001 |
| Number of Bedroom  ≤ 3 Bedrooms  > 3 Bedrooms | 1006 (55.8)  797 (44.2) | 1871 (67.4)  905 (32.6) | <0.0001 |
| **Household indoor environment** |  |  |  |
| Air Conditioning |  |  |  |
| Yes | 588 (32.6) | 1355 (49.0) | <0.0001 |
| No | 1214 (67.4) | 1408 (51.0) |  |

| Dampness in Home |  |  |  |
| --- | --- | --- | --- |
| Yes | 379 (21.1) | 484 (17.5) | 0.003 |
| No | 1419(78.9) | 2274 (82.5) |  |
| Mildew/musty odour |  |  |  |
| Yes | 356 (20.4) | 393 (14.5) | <0.0001 |
| No | 1387 (79.6) | 2314 (85.5) |  |
| Pets in Home |  |  |  |
| Yes | 1027 (56.8) | 1561 (56.1) | 0.644 |
| No | 781 (43.2) | 1221 (43.9) |  |
| Pesticides applied inside Home |  |  |  |
| Yes | 462 (25.8) | 473 (17.3) | <0.0001 |
| No | 1330 (74.2) | 2265 (82.7) |  |
| Use of Tobacco in Home |  |  |  |
| Cigarettes |  |  |  |
| Yes | 220 (12.2) | 476 (17.2) | <0.0001 |
| No | 1582 (87.8) | 2284 (82.8) |  |
| **Household socioeconomic** |  |  |  |
| Total Household Income |  |  |  |
| < $20, 000 | 131 (8.8) | 345 (14.2) | <0.0001 |
| $20,000- $ 39,999 | 287 (19.3) | 572 (23.5) |  |
| $40,000- $ 59,999 | 291 (19.5) | 529 (21.8) |  |
| ≥ $60,000 | 780 (52.4) | 983 (40.5) |  |
| Household Income Adequacy |  |  |  |
| Lowest Income | 66 (4.5) | 173 (7.2) | <0.0001 |
| Lowest Middle Income | 238 (16.0) | 533 (22.2) |  |
| Upper Middle Income | 463 (31.2) | 819 (34.0) |  |
| Highest Income | 716 (48.3) | 881 (36.6) |  |
| Money Left at End of Month |  |  |  |
| Some money | 957 (59.7) | 1471 (57.8) | 0.009 |
| Just enough money | 318 (19.8) | 604 (23.7) |  |
| Not enough money | 328 (20.5) | 470 (18.5) |  |
| **Household Access to Health Care in the past 12 months** |  |  |  |
| Access to family doctor (nurse practitioner) |  |  |  |
| Yes | 1646 (92.0) | 2519 (91.8) | 0.835 |
| No | 143 (8.0) | 224 (8.2) |  |
| Difficulty obtaining primary care |  |  |  |
| Yes | 337 (19.1) | 489 (17.9) | 0.298 |
| No | 1429 (80.9) | 2250 (82.1) |  |
| Required medical specialist care |  |  |  |
| Yes | 1037 (58.1) | 1703 (62.4) | 0.004 |
| No | 747 (41.9) | 1027 (37.6) |  |
| Difficulty obtaining specialist care |  |  |  |
| Yes | 267 (26.1) | 376 (22.1) | 0.019 |
| No | 756 (73.9) | 1322 (77..9) |  |
| Required 24 hr emergency care |  |  |  |
| Yes | 244 (20.2) | 426 (22.4) | 0.161 |
| No | 962 (79.8) | 1480 (77.6) |  |
| Difficulty obtaining 24 hr emergency Care |  |  |  |
| Yes | 37 (14.7) | 83 (18.8) | 0.166 |
| No | 215 (85.3) | 358 (81.2) |  |
